# B Cell Lymphocytosis in Juvenile Dermatomyositis

**DOI:** 10.3390/diagnostics13162626

**Published:** 2023-08-08

**Authors:** Christopher Costin, Amer Khojah, Elisa Ochfeld, Gabrielle Morgan, Saravanan Subramanian, Marisa Klein-Gitelman, Xiao-Di Tan, Lauren M. Pachman

**Affiliations:** 1 Division of Pediatric Rheumatology, Ann & Robert H. Lurie Children’s Hospital of Chicago, Chicago, IL 60611, USA; 2 Department of Pediatrics, College of Medicine, Umm Al-Qura University, Makkah 24341-6660, Saudi Arabia; 3 Division of Allergy and Immunology, Children’s Hospital of Philadelphia, Philadelphia, PA 19104, USA; 4 Department of Pediatrics, College of Medicine, University of Illinois Chicago, Chicago, IL 60612, USA

**Keywords:** B cell, BAFF, neopterin, juvenile dermatomyositis, T cell, biomarkers

## Abstract

In this study, we determined if B lymphocytosis may serve as a JDM biomarker for disease activity. Children with untreated JDM were divided into two groups based on age-adjusted B cell percentage (determined through flow cytometry): 90 JDM in the normal B cell group and 45 in the high B cell group. We compared through *T*-testing the age, sex, ethnicity, duration of untreated disease (DUD), disease activity scores for skin (sDAS), muscle (mDAS), total (tDAS), CMAS, and neopterin between these two groups. The patients in the high B cell group had a higher tDAS (*p* = 0.009), mDAS (*p* = 0.021), and neopterin (*p* = 0.0365). Secondary analyses included B cell values over time and BAFF levels in matched patients with JM (juvenile myositis) and concurrent interstitial lung disease (ILD); JM alone and healthy controls Patient B cell percentage and number was significantly higher after 3–6 months of therapy and then significantly lower on completion of therapy (*p* =< 0.0001). The JM groups had higher BAFF levels than controls 1304 vs. 692 ng/mL (*p* = 0.0124). This study supports B cell lymphocytosis as a JDM disease-activity biomarker and bolsters the basis for B cell-directed therapies in JDM.

## 1. Introduction

Juvenile dermatomyositis (JDM) is a rare systemic pediatric autoimmune disease characterized by skin rash and muscle inflammation [1]. The disease remains heterogenous with many varying phenotypes. Patients with JDM may have presentations ranging from skin-limited disease (JDM sine myositis) to severe presentations including vasculitic skin disease and bowel involvement to unique phenotypes such as MDA-5-associated JDM or antisynthetase syndrome [1,2]. Both typical JDM and its unique phenotypes are thought to be driven by autoantibodies [3,4]. B cells remain a critical element in JDM pathophysiology, both through the production of pathogenic autoantibodies but also as a target for the treatment. [5]. Many JDM treatments, including methotrexate, corticosteroids, mycophenolate, or rituximab, directly or indirectly target B cells [1]. Prior studies have shown that IgG level may be associated with systemic lupus erythematosus (SLE) or Sjogren disease activity; in this study we evaluate general B cell lymphocytosis as a biomarker for disease activity in juvenile dermatomyositis [6].

B cells have been evaluated in JDM as part of the rituximab in myositis (RIM) trial. In this trial, patients with JDM were treated with rituximab, and had an expected decrease in their B cells after treatment [7]. The trial found that 83% of the refractory myositis patients achieved a definition of improvement after receiving rituximab [7]. An analysis of the RIM patient population found that those myositis patients with positive myositis specific antibodies (i.e., anti-Jo-1, anti-Mi-2) responded more favorably to rituximab [8]. B cells have been directly investigated in JDM. One study found that CD19+CD24hiCD38hi immature transitional B cells are elevated in untreated JDM, and that these B cells correlate with JDM disease activity [9]. These findings provide evidence that B cells are pathogenic in JDM and lend credence to the hypothesis that general B cell lymphocytosis may serve as a readily available JDM biomarker for disease activity. In other autoimmune diseases such as rheumatoid arthritis, B cells are routinely monitored longitudinally, usually as a facet of rituximab therapy [10]. Reconstitution of B cells may be a harbinger of disease activity and often elicit further therapy in these disease populations [10].

Our objectives in this study were to establish whether B cell lymphocytosis, determined through flow cytometry as high B cell percentage for age at diagnosis, may serve as a JDM biomarker through comparing the disease characteristics of JDM patients with high numbers of B cells versus JDM with normal B cell percentages. We also explored the associations of absolute B cell number and JDM activity. Other secondary objectives in this study were evaluating the change in B cells during treatment duration and the association of BAFF with juvenile myositis disease activity.

BAFF regulates B cell homeostasis and drives B cell production [11]. BAFF is known to be dysregulated in SLE and is even a target of therapy in SLE through the drug belimumab [12]. Although distinct and separate diseases, SLE and JDM share multiple features, including autoantibodies, photosensitive rashes, arthritis, and myositis [1,13,14]. JDM and SLE are both driven by type I interferons and may have similar pathways of immune dysregulation [12,13]. BAFF levels have previously been analyzed in a cohort of mostly MDA-5 positive JDM patients in Japan where BAFF levels were found to be high, compared to controls, and to be correlated with the presence of rapidly progressive interstitial lung disease (ILD) [15]. BAFF is also associated with the presence of ILD in combined variable immunodeficiency [16]. A secondary goal of this is to investigate BAFF levels in a subset of juvenile myositis (JM) patients with and without ILD compared to matched controls to determine whether BAFF levels correlate with the presence of ILD in JM and to determine if BAFF may also serve as a biomarker for general JM disease activity.

## 2. Materials and Methods

### 2.1. Study Design and Population

This study was performed as a retrospective chart review using the Cure JM biorepository and registry. All patients diagnosed with JM that were seen at Lurie Children’s Hospital were invited to enroll in this registry via written informed consent. Inclusion criteria for the secondary data analysis portion of the study included all children seen at Lurie Children’s between 1980 and 2021, who met the Bohan and Peter criteria for JDM, and who had treatment-naive flow cytometry data [17,18]. For this initial analysis, patients with a diagnosis of overlap syndrome (such as positive anti-U1 RNP, anti-U2 RNP, or anti-PM-Scl with features of autoimmune disease other than JDM) were excluded. Clinical and laboratory parameters were compared between the groups. Healthy controls were utilized in the second analysis within this study and were recruited from allergy clinic or by flyer.

### 2.2. Data Collection and Laboratory Analysis

For all patients included in the study, we obtained their available laboratory data and clinical evaluations at the time of JDM diagnosis that were included in the Cure JM biorepository and registry. Clinical parameters included the total disease activity score (tDAS), skin disease activity score (sDAS), muscle disease activity score (mDAS), Childhood Myositis Assessment Scale (CMAS), nailfold capillary end row loops density per mm (ERL), and duration of untreated disease (DUD). The disease activity score is a scale used to determine disease activity-based observations of skin and muscle disease. The tDAS is a composite score ranging from 0 to 20 that consists of the skin disease activity score (sDAS) ranging from 0 to 9 and the muscle disease activity score (mDAS) ranging from 0 to 11 [19]. The CMAS is a measure of strength that was independently assessed by a certified physical therapist [20,21]. ERL was assessed by the use of combined camera and dermatoscope and was determined by averaging the number of end-row capillary loops per mm in the eight digits, excluding thumbs [22]. DUD is computed in months as time from first reported symptom by the patient to time of initial treatment.

Laboratory parameters included muscle enzymes, T and B cell flow cytometry, inflammatory markers, and myositis-specific antibody testing. Muscle enzymes were measured before treatment and at every visit: creatine phosphokinase (CK), lactate dehydrogenase (LDH), aspartate aminotransferase (AST), and aldolase. Other laboratory parameters included erythrocyte sedimentation rate (ESR), serum neopterin, and von Willebrand factor antigen (vWF) as indicators of disease activity. Serum neopterin, a marker of macrophage activation, was analyzed using a competitive enzyme-linked immunosorbent assay [23]. Clinical laboratory testing was performed at Lurie Children’s clinical and diagnostic laboratory. Flow cytometry was performed to assess T cells, B cells, and natural killer (NK) cells using various antibodies manufactured by BD Biosciences (Franklin Lakes, NJ, USA) to identify CD45, CD3, CD4, CD8, CD16, CD56, and CD19. Flow cytometry was performed by the Lurie Children’s Clinical Immunology Laboratory with B cell reference ranges (as reported below in Supplemental Table S1).

### 2.3. Secondary Analysis

For the secondary analysis of B cells over time, flow cytometry data were obtained three times: at diagnosis, 3–6 months after diagnosis, and on completion of the steroid therapy. Myositis-specific antibodies (MSA) were evaluated via immunoprecipitation and immunodiffusion for each patient at diagnosis and performed by the Oklahoma Medical Research Foundation [24].

For the BAFF subanalysis, patients with JDM or overlap JM and ILD, enrolled in the CureJM repository and registry were queried. ILD lung disease is rare in JDM, occurring in 8% of cases. For the BAFF subanalysis, we included patients with overlap juvenile myositis, defined as meeting the Peter and Bohan criteria for JDM but also having features of other autoimmune diseases [2,25]. Patients with ILD that had samples after the diagnosis of ILD were included (n = 13). These patients were age–sex-matched with patients diagnosed with JDM or overlap myositis without ILD, as well as healthy controls. ILD was defined as either a clinical diagnosis of ILD by a board-certified pediatric rheumatologist or immunologist, or CT findings of ILD. Patients who had received rituximab prior to sample availability were excluded. These laboratory and clinical parameters were compared between the groups. BAFF levels were measured from stored serum samples (stored at −80 °C) using a Bio-techne BAFF/BLyS Elisa kit.

### 2.4. Ethical Statement

This study was performed in accordance with the Declaration of Helsinki. This study was reviewed and approved by the Ann & Robert H. Lurie Children’s Hospital of Chicago, IRB 2010-14117 and IRB 2008-13457 with yearly renewed approvals. Healthy controls were utilized in the second analysis within this study and have given written informed consent per Lurie Children’s IRB 2001-11715.

### 2.5. Statistical Analysis

The JDM patients were stratified into a high B cell percentage group and a normal B cell percentage group. The high B cell group was defined as having an elevated B cell percentage for age with the normal B cell group defined as having normal B cell percentage for age, as defined in Supplemental Table S1. The statistical software GraphPad Prism (version 9.4.1) was used to perform unpaired T testing to compare the baseline characteristics, laboratory parameters, and clinical characteristics between groups. Fisher exact testing was used to analyze the racial backgrounds and MSA differences between the groups. The elevated and normal B cell groups were compared by using *t*-tests. Pearson correlation testing was used to test the correlation of disease parameters and B cell count. Mann–Whitney testing was used to compare BAFF levels between the JM with and without ILD and control groups. Spearman correlation was used to assess the correlation between BAFF levels and disease parameters. Paired *t*-tests were used to compare B cells during the treatment duration.

## 3. Results

A total of 135 children with untreated JDM (75% female, 25% male) were included in the B cell comparative analysis. The racial background and MSA distribution are shown in Table 1. Among the 135 patients, 90 patients had normal B cell percentage for age at diagnosis with 45 patients having high B cell percentage for age at diagnosis. There were no statistically significant differences between demographics of the elevated and normal B cell JDM groups. The MSA distributions between the groups were not significantly different. MSA data were available for 91% (41/45) of the elevated B cell group and 67% (61/90) of the normal B cell group. A subanalysis showed that the elevated B cell group had a moderately higher number of anti-MJ-positive JDM patients with six in the elevated B cell group and one in the normal B cell group (chi-square *p* = 0.003).

As seen in Table 2, the elevated B cell group also had elevated markers of disease activity, including neopterin, tDAS, and mDAS. The ERLs were significantly lower in the elevated B cell count JDM group (*p* = 0.04). Spearman correlation (Table 3) testing of the 135 JDM patients showed that the B cell count significantly correlated with neopterin and inversely correlated with CK, AST, and LDH. Clinically, the B cell count was also significantly correlated with the duration of untreated disease and inversely correlated with CMAS and nailfold capillary end row loops (Table 3).

**Figure 1:**
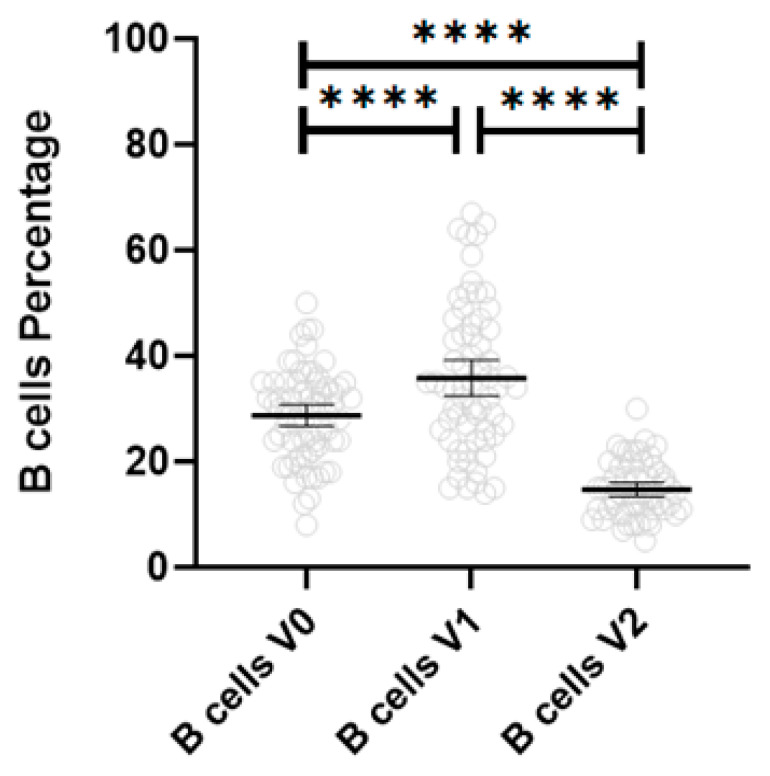
B Cell percentage over time in juvenile myositis. V0 = prior to treatment, V1 = after 3–6 months of therapy, V2 = completion of steroid therapy; **** = *p* < 0.0001.

Subanalysis of the patients with 60-month follow-up data (n = 69) gave results documenting that the B cell percent was significantly elevated 3–6 months after treatment, from 28.7% ± 8.5 (mean ± std. dev) to 35.8% ± 13.7 (*p* =< 0.0001), and then significantly decreased at the completion of therapy to 14.7% ± 5.1 (*p* =< 0.0001), as seen in Figure 1.

**Figure 2:**
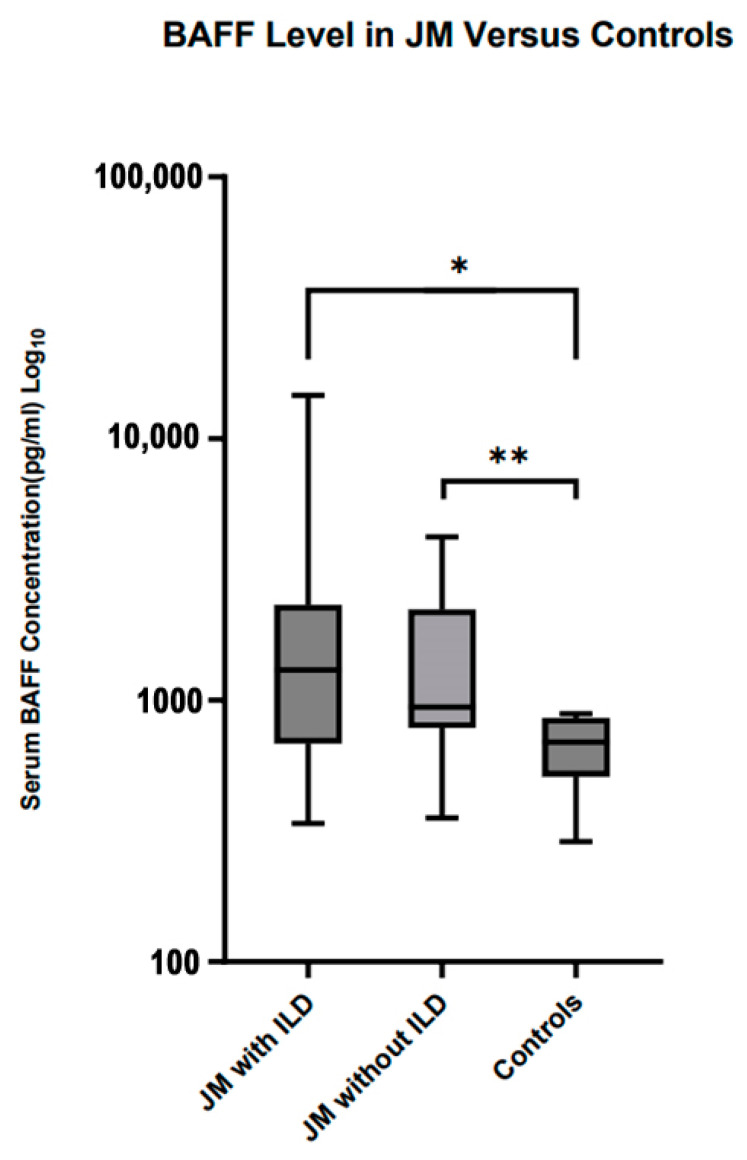
BAFF levels in juvenile myositis patients during disease versus controls; * = *p* < 0.05, ** = *p* < 0.01.

BAFF levels were assessed in 26 JM patients, of which 13 had ILD. BAFF levels were also measured for 13 healthy controls. Demographics and MSA distribution for these groups are shown in Table 4. The BAFF levels were significantly elevated in the 13 JM patients with ILD compared to healthy controls (median 1304 pg/mL IQR 1360 pg/mL vs. 692 pg/mL IQR 481 pg/mL *p* = 0.0124) and in the 13 JM patients without ILD (median 940 pg/mL IQR 481 pg/mL vs. 692 pg/mL IQR 279 pg/mL *p* = 0.0061) when compared to controls, as shown in Figure 2. There were no significant differences in BAFF levels between the JM with ILD and JM without ILD groups (median 1304 pg/mL vs. 940 pg/mL *p* = 0.76). Spearman correlation testing was utilized to analyze the relationship between BAFF levels and disease parameters. BAFF level significantly inversely correlated with absolute lymphocyte count (−0.83, *p* = 0.008) and total cytotoxic T cells (−0.56, *p* = 0.003), as shown in Table 5.

## 4. Discussion

This study demonstrates the relationship between high B cell count for age at diagnosis and disease activity in children with untreated JDM. Patients with elevated B cell count at diagnosis had increased disease activity markers, such as neopterin, total disease activity score (tDAS), muscle weakness disease activity score (mDAS), and were associated with anti-MJ positivity.

B cells have long been implicated in the pathogenesis of autoimmune diseases. The immune pathophysiology of JDM remains complex, driven directly by autoantibodies but also through other immune pathways including the interaction with T cells and the innate immune system [1]. Presentations of JDM remain broad with phenotypes ranging from unique phenotypes such as anti-synthetase syndrome to skin-limited disease, and to a more vasculitic presentation with bowel disease and vasculitic rash [1].

A wide variety of biomarkers and clinical features are used to assess JDM disease activity [26]. New biomarkers of disease activity are needed to aid in monitoring JDM treatment and to possibly predict outcomes and treatment response. We found that the elevated B cell group, on the whole, had higher disease activity involving both clinical and laboratory features. The elevated B cell group had higher tDAS and mDAS consistent with worsened clinical disease. The CMAS was lower in the elevated B cell group, although this did not reach significance. On correlation testing, the CMAS significantly inversely correlated with B cell count, further bolstering the association of B cell count and more extensive muscle weakness. Patients in the elevated B cell group had lower ERL counts. Nailfold ERLs reflect vasculopathy and also support worsened clinical disease in the elevated B cell group. Correlation testing showed that as B cells rose, the neopterin and ERLs worsened, supporting the association of elevated B cell count with worse disease activity. Overall, children with JDM in the elevated B cell group had clinically worse disease, thus supporting the use of B cell count as biomarker for disease activity.

Neopterin is a byproduct of macrophage activation and is a known biomarker of JDM activity [23]. We found that the high B cell group had significantly elevated neopterin levels, compared to the normal B cell group. Neopterin correlated with B cell count among the JDM patients. The elevated B cell group had significantly different flow cytometry results with lower total T-cells, T helper cells, and NK cells. NK cells are an emerging biomarker of JDM activity. Low NK cell count in the high B cell group is further evidence of B cell count as a marker of JDM activity [27]. The total T cell and T helper cell populations were significantly different between the high and normal B cell groups; however, these T cell populations remained in the normal range for both groups. The importance of the difference between the groups remains unknown and bears further investigation.

T cells are dysregulated in JDM. However, this dysregulation has been better described in the T-reg cell and Th17 cell populations [28,29]. T cells are found in JDM muscle biopsies, and we hypothesize that the elevated disease activity observed in the high B cell group possibly associates with or drives T cells into muscle tissues, although this requires further investigation [30].

Other findings include a correlation of higher B cells with lower muscle enzymes and vWF (a known JDM biomarker) [31]. This may in part be explained by a longer duration of untreated disease in the high B cell group. Chronic uncontrolled myositis leads to replacement of muscle with fat and scar tissue, leading to lower muscle mass and may result in a gradual decrease in muscle enzymes despite ongoing active myositis [32,33].

Examination of the MSAs-untreated children with JDM in the high and normal B cell groups on the whole resulted without significant differences beyond an increased number of anti-MJ-positive (Anti-Nxp2) patients in the high B cell group. Anti-MJ-positive JDM has been shown to have increased serum neopterin and lower CMAS [23]. The finding that the high B cell group had more anti-MJ-positive patients supports that the notion that anti-MJ-positive JDM may be a unique phenotype that may be more B cell-based. This finding provides a theoretical rational for the use of B cell-aimed agents in this, especially in this JDM subset.

Our subanalysis, for patients with available follow-up data, showed that B cells of JDM patients first increased with therapy and then eventually subsided to a lower percentage of the total lymphocytes. This is likely multifactorial, with the increased B cell percentage during therapy in part reflecting the known effect of steroids decreasing peripheral T cell populations and causing a relative B cell lymphocytosis [34]. The lower B cells on completion of therapy may suggest resolution of immune dysregulation, but also to the withdrawal of any drug effect. Since elevated B cells associate with markers of JDM disease activity, a persistent B cell lymphocytosis, especially on withdrawal of steroids, may indicate ongoing JDM activity and possibly treatment resistance, although this bears further study.

BAFF is a cytokine known to drive B cell proliferation and homeostasis [11], and we found that BAFF was significantly elevated in the JM patients compared to controls. A prior study in a Japanese JDM population showed that BAFF levels were associated with rapidly progressive ILD [15]. We did not find a significant difference between the BAFF levels among the JM with ILD and JM without ILD, and this may in part be explained by a higher rate of MDA5 positivity in the Japanese study. Correlation testing showed that BAFF levels inversely correlated with absolute lymphocyte count and cytotoxic T cells. BAFF has been shown to stimulate both cytotoxic and helper T cell populations [35]. BAFF has been shown to cause IL2 and IL2-R expression [35]. With this in mind, it remains possible that BAFF may in part be a factor in macrophage phage activation syndrome related to JDM or broader T cell dysregulation. It is also possible that elevated BAFF may help drive CD8-positive T cells into muscle tissue, accounting for the inverse correlation, although this requires further investigation [35]. Although BAFF was inversely correlated with a low absolute lymphocyte count, it was not associated with a change in B cell populations. This may be due to the small nature of our subanalysis but also may suggest multiple drivers of B cell production.

In summary, we found that JDM patients with elevated B cells had worse JDM, as indicated by both laboratory and clinical parameters. B cell count correlated with clinical and laboratory markers of the disease. Overall, we found that B cell lymphocytosis serves as a biomarker for disease activity in JDM. Our subanalysis showed that B cell lymphocytosis increased during therapy but resolved on treatment, further supporting the use of B cells as a biomarker in JDM. As opposed to prior studies, we did not find BAFF levels to be significantly higher in JM-associated ILD compared to typical JM. We found that BAFF was elevated in JM, as compared to healthy controls, although it did not associate with clinical or laboratory markers of disease activity, making it a poor biomarker. Elevated BAFF and B cells in JDM support B cell-directed therapies in JDM. Of particular interest is the BAFF monoclonal antibody belimumab.

Limitations of this study include that it was not powered to determine subtle differences between MSA subgroups of JDM. Furthermore, this study looked primarily at B cells during JDM diagnosis and does not comment on B cells and their association with other biomarkers and clinical markers of JDM disease activity through time. We found that B cells increased during therapy and then subsided on completion of therapy, but we were unable to determine whether B cells predict disease outcome. We propose that future studies be aimed at predicting JDM outcomes consider B cells or even BAFF levels as predictors of disease outcome. Despite finding that the high B cell group had a significant increase in many JDM disease biomarkers (e.g., tDAS, neopterin), some were only slightly elevated in the high B cell group and are of unclear clinical importance. Our BAFF analysis was also limited by a small sample size and may have been unable to parse out subtle associations with BAFF and other disease markers.

## 5. Conclusions

We found that B cell count may serve as a readily available biomarker for JDM. B cell count increased during the initial phase of therapy and then decreased on remission of JDM. Although B cells proved to be a biomarker of JDM disease activity, their full prognostic value remains somewhat unknown and further studies are needed. Care should be taken in interpretation as normal B cell concentrations change with age. We found that although elevated in JM, BAFF levels did not correlate with typical clinical and laboratory markers of disease. On the whole, this study shows that B cells associate with JDM disease activity and supports B cell-directed therapies in JDM.

## Figures and Tables

**Table 1:** Demographics of the 135 JDM patients.

	High B Cell	Normal B Cell	*p*-Value
Number of subjects (%)	45 (33.3%)	90 (66.7%)	
Age at onset, mean years (±stdv)	6.4 (±3.5)	7.1 (±3.8)	0.27
**Sex, n (%)**			
Female	32 (71.1%)	70 (77.8%)	0.40
Male	13 (28.9%)	20 (22.2%)	
**Race/ethnicity, n (%)**			
White, Non-Hispanic	28 (62.2%)	70 (77.8%)	0.23
White, Hispanic	12 (26.7%)	15 (16.7%)	
African American	1 (2.2%)	2 (2.2%)	
Asian	3 (6.6%)	1 (1.1%)	
Other/Unspecified	1 (2.2%)	2 (2.2%)	
**MSA, n (%)**	**n = 41**	**n = 61**	
P155/140 (Anti-TIF1-γ)	17 (41%)	29 (39%)	0.21
MJ (Anti-NXP-2)	6 (15%)	1(1.3%)	
Mi2	2 (4.8%)	9 (12%)	
MDA5 (anti-CADM140)	1 (2.4%)	2 (2.6%)	
Other MSA	0 (0%)	1 (1.3%)	
Multiple MSAs	3 (7.3%)	6 (8.0%)	
Negative	12 (29%)	13 (37%)	

**Table 2:** High and normal B cell groups: disease activity markers, clinical and laboratory findings.

Flow Cytometry (Cells/μL)	Reference Range	High B Cells (Mean ± S.D.)	Normal B Cells (Mean ± S.D.)	*p*-Value
Total T cells (CD3+)	895–8192	1398 ± 556	1782 ± 974	**0.005**
T helper cells (CD3+ CD4+)	488–4552	929 ± 376	1209 ± 670	**0.003**
T cytotoxic cells (CD3+ CD8+)	270–3749	452 ± 212	529 ± 309	0.15
NK cells (CD16+/CD56+)	121–1581	134 ± 91	184 ± 151	**0.02**
**Laboratory Findings**	**Reference Range**	**High B Cells (Mean ± S.D.)**	**Normal B Cells (Mean ± S.D.)**	***p*-Value**
ESR (mm/h)	0–20	20.89 ± 15.5	16.08 ± 10	0.17
Neopterin (nmol/L)	0–10	22.91 ± 11.23	18.03 ± 11.1	**0.025**
vWF (%)	36–241	149.9 ± 59.4	161.5 ± 88.5	0.46
CK (IU/L)	26–279	737 ± 1634	1912 ± 4386	**0.03**
AST (IU/L)	18–65	79.4 ± 64	111 ± 151	0.20
LDH (IU/L)	147–438	432 ± 185	444 ± 326	0.80
Aldolase (U/L)	3.4–11.8	15.6 ± 14	20.4 ± 22	0.40
**Clinical Findings**	**Testing Range**	**Elevated B Cells (Mean ± S.D.)**	**Normal B Cells (Mean ± S.D.)**	***p*-Value**
tDAS	0–20	11.99 ± 2.88	10.44 ± 3.53	**0.008**
sDAS	0–9	5.98 ± 1.34	5.73 ± 1.31	0.33
mDAS	0–11	6.01 ± 2.40	4.70 ± 3.09	**0.008**
CMAS	0–52	30.35 ± 13.55	35.72 ± 11.37	0.12
ERL/mm	Normal ≥ 7	4.59 ± 1.53	5.26 ± 1.68	**0.04**
DUD	N/A	10.68 ± 12.16	7.59 ± 8.30	0.13

**Table 3:** Correlation coefficients for B cell count and disease parameters in 135 untreated JDM patients.

Laboratory Findings	Spearman’s R	*p*-Value
ESR (mm/h)	−0.14	0.17
Neopterin (nmol/L)	0.22	**0.02**
vWF(%)	−0.39	**<0.001**
CK (IU/L)	−0.23	**0.01**
AST (IU/L)	−0.28	**0.003**
LDH (IU/L)	−0.22	**0.02**
Aldolase (U/L)	−0.06	0.53
**Clinical Findings**	**Spearman’s R**	***p*-Value**
tDAS	−0.14	0.13
sDAS	0.02	0.82
mDAS	−0.15	0.08
CMAS	−0.27	**0.04**
DUD	0.28	**0.002**
ERL/mm	−0.25	**0.012**

**Table 4:** Demographics of 26 JM patients and healthy controls.

	JM with ILD	JM without ILD	Controls	*p*-Value
Number of subjects	13	13	13	
**Sex, n (%)**				
Female	10 (76.9%)	10 (76.9%)	10 (76.9%)	1.00
Male	3 (23.1%)	3 (23.1%)	3 (23.1%)	
**Race/ethnicity, n (%)**				
White, Non-Hispanic	7 (53.8%)	9 (69.2%)	6 (46.1%)	0.94
White, Hispanic	4 (30.7%)	3 (23.1%)	5 (38.4%)	
African American	1 (7.6%)	0 (0.0%)	1 (7.6%)	
Asian	1 (7.6%)	1 (7.6%)	1 (7.6%)	
**MSA, n (%)**	n = 12	n = 13		
P155/140 (Anti-TIF1-γ)	1 (7.7%)	6 (46.2%)		0.43
JO-1	3 (23%)	0 (0%)		
PMSCL+	2 (15.3%)	1 (7.7%)		
MDA5 (anti-CADM140)	1 (7.7%)	0 (0%)		
Other MSA	3 (23%)	3 (23%)		
Negative	2 (15.3%)	3 (23%)		

**Table 5:** Spearman Correlation Coefficients of for BAFF in disease parameters in 26 JM Patients.

Laboratory Findings	Spearman’s R	*p*-Value
ESR (mm/h)	0.33	0.12
Neopterin (nmol/L)	0.40	0.06
CK (IU/L)	0.20	0.37
AST (IU/L)	−0.21	0.025
Absolute Lymphocyte Count	−0.833	**0.008**
Total T Cells (CD3+) count	−0.35	0.08
T Helper Cells (CD3+ CD4+) count	−0.06	0.53
T Cytotoxic Cells (CD3+ CD8+) count	−0.56	**0.003**
NK Cells (CD16+ CD56+) count	−0.21	0.29
B Cells (CD19+) count	−0.16	0.43
**Clinical Findings**	**Spearman’s R**	***p*-Value**
tDAS	0.28	0.17
sDAS	0.27	0.18
mDAS	0.13	0.54
CMAS	−0.18	0.41
DLCO adjusted for ventilation	−0.19	0.26
ERL/mm	−0.27	0.26

## Data Availability

Restrictions apply to the availability of these data. Data were obtained from Cure JM biorepository and Cure JM registry and are available on request: coebiorepository@luriechildrens.org.

## Supplementary Materials

The following supporting information can be downloaded at: https://www.mdpi.com/article/10.3390/diagnostics13162626/s1, Table S1: B cell Subset Reference Ranges [36].
