# Supplementary material for: B Cell Lymphocytosis in Juvenile Dermatomyositis
Source: Diagnostics (Basel). 2023 Aug 8;13(16):2626. doi: 10.3390/diagnostics13162626 (PMC10453137; doi:10.3390/diagnostics13162626)
Supplement: Supplementary file 1 [file diagnostics-13-02626-s001.zip › Supplementary Table S1.pdf]

B cell Subset Reference Ranges
O'Gorman et. al. Cytometry 1999;34:235-241
REFERENCE RANGE

Days of Life	B Cell Percentage	B Cell Absolute Count
124-335	16-36	858-3774
336-690	16-33	704-2711
691-1114	17-34	523-1779
1115-1528	17-37	397-1539
>1528 days	9-32	203-1139
